# An innovative sequential mixed-methods approach to evaluating clinician acceptability during implementation of a standardized labor induction protocol

**DOI:** 10.1186/s12874-023-02010-7

**Published:** 2023-08-29

**Authors:** Rebecca Feldman Hamm, Lisa D. Levine, Julia E. Szymczak, Samuel Parry, Sindhu K. Srinivas, Rinad S. Beidas

**Affiliations:** 1 Division of Maternal-Fetal Medicine, Department of Obstetrics & Gynecology, University of Pennsylvania Perelman School of Medicine, 3400 Spruce Street, 2 Silverstein, Philadelphia, PA 19104 USA; 2 Leonard Davis Institute of Health Economics, University of Pennsylvania Perelman School of Medicine, Philadelphia, PA USA; 3 Department of Internal Medicine, University of Utah, Salt Lake City, UT USA; 4 Department of Medical Social Sciences, Northwestern University Feinberg School of Medicine, Chicago, IL USA

**Keywords:** Acceptability, Acceptability of intervention measure, Hybrid trial, Mixed-methods, Standardization

## Abstract

**Background:**

Implementation outcomes, including acceptability, are of critical importance in both implementation research and practice. The gold standard measure of acceptability, Acceptability of Intervention Measure (AIM), skews positively with a limited range. In an ongoing hybrid effectiveness-implementation trial, we aimed to evaluate clinician acceptability of induction standardization. Here, we describe an innovative mixed-methods approach to maximize the interpretability of the AIM using a case study in maternal health.

**Methods:**

In this explanatory sequential mixed methods study, we distributed the validated, 4-question AIM (total 4–20) to labor and delivery clinicians 6 months post-implementation at 2 sites (Site 1: 3/2021; Site 2: 6/2021). Respondents were grouped by total score into tertiles. The top (“High” Acceptability) and bottom (“Low” Acceptability) tertiles were invited to participate in a 30-minute semi-structured qualitative interview from 6/2021 to 10/2021 until thematic saturation was reached in each acceptability group. Participants were purposively sampled by role and site. Interviews were coded using an integrated approach, incorporating a priori attributes (Consolidated Framework for Implementation Research constructs) into a modified content analysis approach.

**Results:**

104 clinicians completed the initial survey; 24 were interviewed (12 “High” and 12 “Low” Acceptability). Median total AIM scores were 20/20 IQR[20–20] in the High and 12.5/20 IQR[11–14] in the Low Acceptability groups. In both groups, clinicians were enthusiastic about efforts to standardize labor induction, believing it reduces inter-clinician variability and improves equitable, evidence-based care. In the Low Acceptability group, clinicians stated the need for flexibility and consideration for patient uniqueness. Rarely, clinicians felt labor induction could not or should not be standardized, citing discomfort with medicalization of labor, and concerns with “bulldozing” the patient with interventions. Suggested strategies for overcoming negative sentiment included comprehensive clinician education, as well as involving patients as active participants in the protocol prenatally.

**Conclusions:**

This study utilized AIM in an innovative sequential mixed-methods approach to characterize clinician acceptability, which may be generalizable across implementation endeavors. By performing this work during a hybrid trial, implementation strategies to improve acceptability emerged (clinician education focusing on respect for flexibility; involving patients as active participants prenatally) for year 2, which will inform future multi-site work.

**Table:** 

**Text box 1.** Contributions to the literature
• Acceptability of Intervention Measure (AIM) scores skew positively, thus limiting how well they inform optimization of implementation approaches.
• This sequential mixed-methods approach is an innovative way to utilize AIM in evaluating clinician acceptability in healthcare innovation.
• In using this methodology to evaluate clinician acceptability of an implementation endeavor to standardize labor induction practices, several implementation strategies to improve acceptability were identified.

## Background

Implementation outcomes are of critical importance in both implementation research and clinical practice. In Proctor’s seminal work, 8 implementation outcomes were defined: acceptability, appropriateness, feasibility, adoption, fidelity, cost, penetration, and sustainability [1]. More recent work has focused on additional outcomes of importance to implementation endeavors: availability, health equity, and scale-up [2]. Historically, *acceptability*, defined as the perception among implementation stakeholders that a given treatment, service, practice, or innovation is agreeable, palatable, or satisfactory, has received much attention as a perceptual implementation outcome that may precede behavioral implementation outcomes [1, 3, 4]. In fact, in a 2015 systematic review, 50 of 104 instruments for measuring implementation outcomes were measures of acceptability [3]. While there are a number of instruments to measure acceptability, one of the most commonly used gold-standard pragmatic approaches, with strong psychometric properties, is the Acceptability of Intervention Measure (AIM) [3, 5]. The AIM is a 4-item measure scored on a 5-point Likert scale (1 = strongly disagree, 5 = strongly agree), then either averaged [total score 1–5] or reported as a total [total score 4–20].

In prior work using the AIM to evaluate acceptability of a variety of healthcare interventions and/or implementation strategies, scores skew positively with a limited range, thus potentially hindering the utility of the assessment to optimize interventions and/or implementation strategies when used alone [6]. For example, work evaluating a communication program for adult child caregivers of parents with a blood cancer utilized the AIM measure. As no available cutoffs for “acceptable” versus “unacceptable” exist, the authors deemed the intervention would be acceptable if participants had mean scores of 4 or higher on the AIM items. The authors met their acceptability goal [7]. In a project evaluating a huddle to review ongoing quality improvement work in a pediatric intensive care unit, AIM was used prior to implementation. Despite lack of a clear cutoff, the authors determined their intervention to be adequately acceptable with a mean total AIM score of 15.2, and moved forward with implementation [8]. Yet, a measure like AIM likely has value beyond developing a cutoff for acceptability, which most interventions would likely meet.

In addition, prior work evaluating acceptability using qualitative methods has generally focused on the perspectives of clinicians thought to be key stakeholders due to their role, limiting the capture of a full range of sentiments [9–11]. One possible method to select interview respondents to obtain richer data involves purposeful sampling using deviant cases [9]. A purposeful sampling approach utilizing deviant cases involves selecting participants at extreme ends of a spectrum in manifestations of belief or behavior around a healthcare intervention for inclusion in a sample for in-depth qualitative study. The logic of purposeful sampling utilizing deviant cases is that the approach facilitates the inclusion of individuals in a sample that manifest the phenomenon of interest with sufficient intensity to produce variation in expression, thus increasing the analytic value of the sample. This is a pragmatic and innovative way to overcome barriers to assembling a sample in a qualitative study of acceptability that contains people whose daily experience will be influenced by healthcare implementation who hold both positive and negative views about the proposed evidence-based practice. In particular, interviewing positive deviants around healthcare implementation, such as those who have been most successful at enacting change, has yielded valuable results. However, this method has not yet been applied to acceptability [10–12].

Here, we describe an innovative explanatory sequential mixed methods approach to evaluate the implementation outcome of acceptability, which harnesses the validity of AIM to provide a deeper look at acceptability. We utilized this method during a 2-year pre- and post-implementation type I hybrid effectiveness-implementation trial incorporating a standardized labor induction protocol into routine care at 2 urban labor and delivery units. The goal of this protocol intervention is to reduce the cesarean rate overall, as well as reduce racial and ethnic disparities in cesarean [13, 14]. We performed this acceptability assessment after one year of implementation to optimize the second year.

## Methods

### Parent hybrid type I effectiveness-implementation trial

This explanatory sequential mixed-methods (QUAN->QUAL) study was embedded into a broader type I hybrid effectiveness-implementation trial evaluating implementation of a standardized protocol for the management of labor induction into routine care at 2 urban labor and delivery units. Site #1 is an academic, high acuity labor and delivery unit delivering approximately 4200 patients per year staffed primarily by obstetrician-gynecologists and maternal-fetal medicine specialists, with associated residency and fellowship programs. Site #1 also has family practice attending physicians, residents, and a small group of midwives. Site #2 is a community based program delivering approximately 5000 patients per year staffed by obstetrician-gynecologists and an associated residency program, as well as a large midwifery program.

The hybrid trial is a 2-year stepped pre- and post-implementation trial, beginning October 1, 2018 and concluding December 31, 2022. The protocol was initiated at Site #1 on October 1, 2020, and at Site #2 on January 1, 2021. The standardized labor induction protocol was approved by a multidisciplinary institutional obstetrical committee prior to its initiation and includes recommendations for frequent cervical exams and interventions such as oxytocin and amniotomy at particular time points, with a focus on active management of labor induction. Implementation strategies for the initial implementation plan included conducting local consensus discussions, conducting educational meetings with clinicians in the 3 months leading up to implementation at each site, and audit and feedback reports, which detailed site-level compliance with the protocol and were distributed to sites every 3 months post-implementation.

The hybrid trial has 2 aims: (1) to determine the effectiveness of implementing a standardized labor induction protocol at improving obstetric outcomes, specifically cesarean delivery rate, and (2) to evaluate implementation outcomes relevant to standardized labor induction protocol (acceptability, penetration, and fidelity) using a mixed-methods approach. Here, we will specifically discuss our methodology and results related to the evaluation of acceptability within the context of the broader trial. The University of Pennsylvania Institutional Review Board approved all study procedures; informed consent was obtained prior to initiation of survey and interview components of the study. SRQR guidelines were utilized in preparing this manuscript.

### Quantitative methods

The validated, 4-item AIM was modified to our intervention ([Triple P/Implementation Strategy] replaced with “the standardized labor induction protocol”) and distributed using clinician listservs 6 months after implementation at each of the 2 participating sites (Site #1: 3/2021; Site#2: 6/2021). In addition to the AIM questions, clinicians were asked demographic items including role, age, gender identity, and years in practice; survey responses were otherwise anonymous. At the completion of the survey, clinicians were asked if they would be willing to participate in the qualitative portion of the study, and, if willing, asked for their name and e-mail address to connect their survey response to identifying information.

After survey closure, AIM scores were totaled and grouped into “tertiles”: highest 1/3 of scores (“High” Acceptability), middle 1/3 of scores (“Middle” Acceptability), and lowest 1/3 of scores (“Low” Acceptability) among all respondents. Tertiles were selected in order to ascertain positive and negative deviants and thus determine who would be the most data rich respondents for the qualitative section of our work. Skew of the AIM total score was determined using skew and kurtosis tests for normality.

### Qualitative methods

**Fig. 1:**
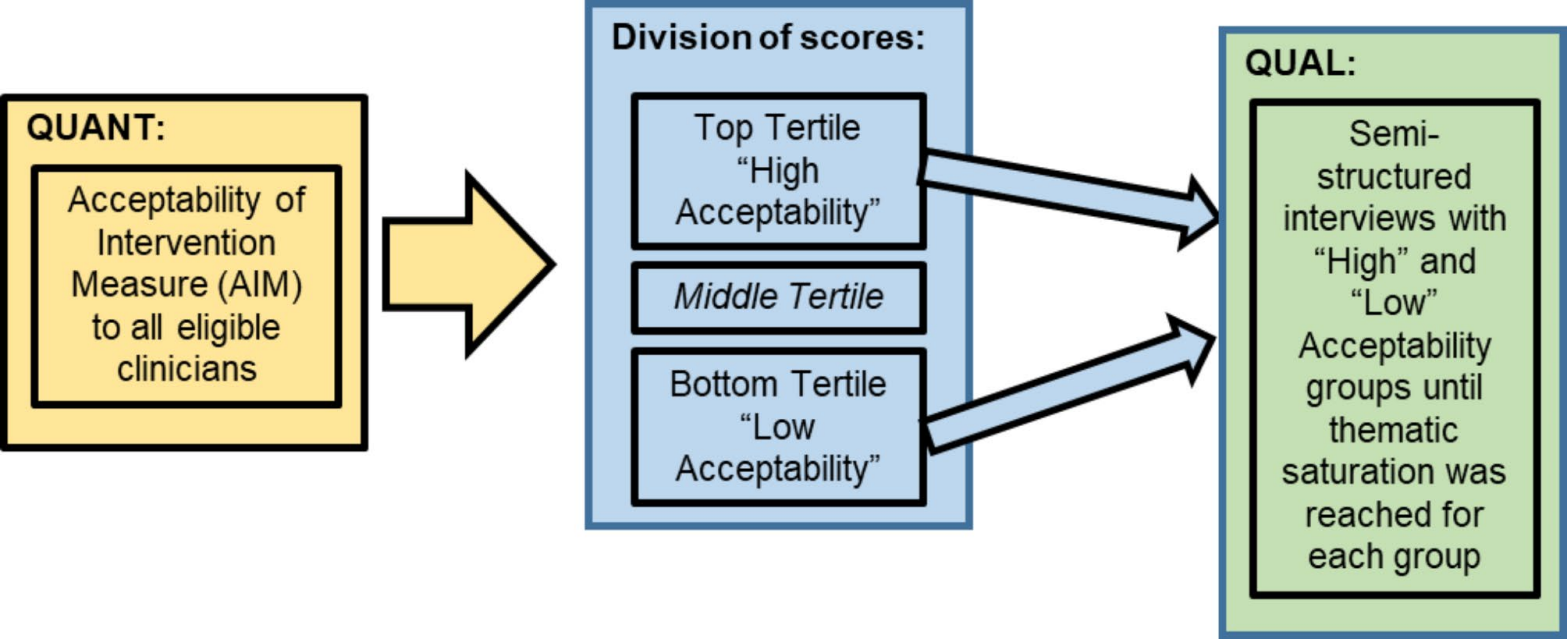
Summary of the quantitative to qualitative approach to acceptability

Clinicians who stated interest in participating were contacted from each of the High and Low Acceptability tertiles, with purposive sampling by clinician role (attending physician, trainee physician, nurse-midwife, nurse) and site until thematic saturation was achieved for both the High and Low Acceptability groups. We monitored for thematic saturation in both acceptability groups by noting the emergence of themes by interview question at the conclusion of each interview. We determined the groups to be saturated when we ceased to observe novel themes in the answers to each interview question. A summary of this QUAN->QUAL approach is shown in Fig. 1.

The Consolidated Framework for Implementation Research (CFIR) was used to create the interview guide [15]. Interview questions elicited (1) acceptability of efforts to standardize labor induction, (2) barriers and facilitators to implementation of standardized labor induction practices generally, (3) acceptability of, as well as barriers and facilitators to implementation of specific components of the labor induction protocol (such as recommendations for frequent cervical exams and interventions like oxytocin and amniotomy at particular time points), (4) acceptability of current implementation strategies around the standardized labor induction protocol (including local consensus discussions, clinician education meetings, and audit and feedback) and suggestions for improved implementation. A summary of interview questions is shown in Table 1. Questions were designed to be open ended.

**Table 1:** Summary of interview questions

**General Questions**	*What do you think about efforts to standardize clinical practices on labor and delivery, in general?*
	*What do you think about efforts to standardize the management of labor induction? Can labor management be standardized?*
	*What do you think about the goal of reducing cesarean rate?*
**Barriers and facilitators to implementation of standardized labor induction practices in general**	*Did you utilize the protocol for patients who qualified? Can you tell me more about the reasons why/why not?*
	*How do you feel the standardized protocol compares to what you did before?*
	*Can you think of barriers that got in the way of using the standardized induction protocol?*
	*What was your experience interacting with other clinicians or staff on your unit in relation to using the induction protocol?*
	*How has the culture of labor and delivery at [insert hospital] influenced the use of the induction protocol?*
	*Are there any external to the hospital factors (national policies or laws) that you think have influenced the implementation of the induction protocol on your unit?*
	*Do you ever have to choose between what safety or quality processes to follow due to time or resource constraints? How does the standardization of labor induction implementation fare when you have to make those kinds of choices?*
**Acceptability of current implementation strategies around the standardized labor induction protocol**	*What are your thoughts about how the evidence behind the induction protocol was described and communicated to you?*
	*We used a few strategies to try to facilitate the implementation of the induction protocol and I would like your opinions about each, especially whether the strategy helped.*
**Suggestions for improved implementation and sustainability**	*What kinds of changes could be made to improve the protocol?*
	*Would additional resources or supports help increase the use of the protocol? If yes, what are they?*
	*Do you plan to use the labor induction protocol moving forward?*
	*Do you think the standardized labor induction protocol will be used by others on your unit long term?*
	*If you went to another institution, would you take it with you as a whole? As specific components?*

Semi-structured interviews occurred for those enrolled from June 3, 2021 to July 31, 2021 for Site #1 participants and September 1, 2021 to October 31, 2021 for Site #2 participants (9–10 months post-implementation at each site). Individual interviews were conducted in-person, via video conferencing software, or over the phone and lasted an average of 30 min. The primary investigator (RFH), an obstetrician trained in qualitative interviewing, conducted all interviews.

Permission was obtained and all interviews were recorded. Audio from the interviews was transcribed by Datagain Transcription Services (Secaucus, NJ). The transcripts were then uploaded to NVivo 12 software for management and coding. A research coordinator trained in qualitative methods used an inductive process of iterative coding to ascertain recurrent relationships, themes, and categories and to develop the codebook. Then, we used an integrated analysis approach [16], identifying a priori attributes (CFIR constructs) and also using modified content analysis approach. Two trained research personnel (MN and MW) applied the codebook to the transcripts and periodically refined the themes and definitions based on inter-rater reliability tests in order to facilitate analysis. 20% of transcripts were double-coded (k = 0.83). The researcher coordinators then synthesized the outputs of the coding and identified the key themes described in this manuscript. Analysis was completed in February 2022.

## Results

### Quantitative results

104 clinicians across both sites completed the AIM survey, 61.5% from Site #1 and 38.5% from Site #2. Clinicians participating in the survey were evenly split among physicians and nurses, were primarily under 45 years of age, and 87.5% identified as female (Table 2).

**Table 2:** Demographic characteristics of participants: (1) Survey participants overall, and (2) Qualitative participants by acceptability group

	Survey (QUAN) participants (n = 104)	High Acceptability QUAL participants (n = 12)	Low Acceptability QUAL participants (n = 12)
Role			
Attending Physician	30 (28.8)	3 (25.0)	4 (33.3)
Resident/Fellow Physician	27 (26.0)	6 (50.0)	2 (16.7)
Certified nurse-midwife	4 (3.8)	0 (0)	2 (16.7)
RN	43 (41.3)	3 (25.0)	4 (33.3)
Age			
≤ 35	57 (54.8)	7 (58.3)	7 (58.3)
36–45	34 (32.7)	4 (33.3)	1 (8.3)
46–55	9 (8.7)	1 (8.3)	2 (16.7)
≥ 56	4 (3.8)	0 (0)	2 (16.7)
Gender			
Female	91 (87.5)	11 (91.7)	11 (91.7)
Male	13 (12.5)	1 (8.3)	1 (8.3)
Years experience in role			
≤ 5	45 (43.3)	6 (50.0)	5 (41.7)
6–10	28 (27.0)	4 (33.3)	3 (25.0)
11–20	22 (21.2)	1 (8.3)	3 (25.0)
> 20	9 (8.7)	1 (8.3)	1 (8.3)

Median total AIM score was 15/20 IQR [12–19], with a notable positive skew (p = 0.03). In determining cut points for tertiles, those with scores ≥ 17 (n = 28) were placed in the “High Acceptability”, 14–16 (n = 33) in the “Middle Acceptability”, and ≤ 13 (n = 43) in the “Low Acceptability” groups. 42/104 (40.4%) of respondents reported willingness to participate in the qualitative aspect of the work at the end of the survey, which were distributed across acceptability groups (High = 17; Middle = 13; Low = 12). Those who agreed to participate in the qualitative work were overall similar in demographic characteristics to those who did not (results not shown).

### Qualitative results

A total of 24 interviews were performed, 12 in the High and 12 in the Low Acceptability groups. Interviewees included 15 physicians (8 trainees and 7 attending level; 13 obstetrician-gynecologists and 2 family medicine physicians), 2 certified nurse-midwives, and 7 registered nurses. Median total AIM scores were 20/20 [IQR 20–20] in the High and 12.5/20 [IQR 11–13] in the Low Acceptability groups, consistent with the positive skew of this value. Characteristics by acceptability group enrolled in the qualitative portion of the study are also shown in Table 2.

#### Acceptability of efforts to standardize labor induction

Broadly, participants reported positive sentiment regarding efforts to standardize obstetric care and to reduce rates of cesarean delivery. Regardless of acceptability group, providers were generally enthusiastic about the induction protocol and efforts to standardize labor induction. Participants cited a range of reasons for valuing standardized labor management, such as feeling that the intervention will make patient care more equitable and reduce variability between clinicians, that they appreciate being guided by evidence-based practices, or that they like having a tool for learning or to refer to if they receive pushback from patients or other providers.

I love it. I find that there is a lot going on the labor floor, and so whenever there’s something [like] a protocol to rely on and actually physically point to, or just have at our disposal is very helpful.

While most participants reported they believed labor could be standardized, those in the Low Acceptability group were more likely to add nuance to this belief, noting that providers also need to be flexible and consider the specific needs of each unique patient.

I think it’s good [to standardize labor practices], I think that aim to make sure that everyone is being cared for on the same level and given the same attention is good. I do think that there has to be room for specifications to the situation and to the patient, and so while it’s good to standardize things, I think you still have to be open to what happens and what changes with the patient specifically.

Occasionally, participants in the Low Acceptability group felt labor induction could or should not be standardized. Those who felt induction should not be standardized cited several reasons: (1) They felt patient education levels about treatment options vary. Clinicians did not always believe patients were given the opportunity to fully learn about intervention steps before being asked to complete one; (2) The perception that labor and delivery should not be medicalized more than is necessary.

…Our clinicians are mostly lovely and are mostly telling women what they’re going to do, asking them if they’re okay to do it. I’ve just had a few incidents recently where I felt like people were being bulldozed a little bit.

I don’t think women are standard. I think it’s very challenging to work with sort of a checklist checkbox model of labor, induction, birth, any of those things. I feel like a lot of our patients feel very vulnerable and almost assaulted by the process and in some ways I feel I’m almost in a position of needing to protect my patients from sometimes excessive intervention and rushing of interventions that they may not be fully educated about or really informed of, or consent to. Like people just walking in the room and saying, “We’re going to break your water now.“

#### Barriers and facilitators to implementation of standardized labor induction practices in general

Participants discussed several key barriers to protocol use, which were primarily focused on the CFIR constructs of Inner Setting (such as lack of adequate staffing or time) and Characteristics of Individuals (including clinician and patient factors).

Almost all participants, regardless of acceptability group, described their floors as being very busy, with high patient volume, or not having sufficient staff to meet all patient needs in a timely manner. Participants tended to say these demands on their or their peers’ time impeded their ability to implement the protocol because they simply needed to be elsewhere, other cases were more urgent and needed to take priority, or that juggling so many tasks led them to forget a protocol step.

[The barrier] is the volume [of patients] and the lack of providers being available at the right time. I feel like as a nurse, there are definitely times– like just yesterday—where…they said [the patient’s next cervical exam should be] around 1:00…And then generally, it’s like, ‘Oh, well, there are a couple of deliveries, but we’ll be in here soon.’ I’m like, ‘You’re on the list and we know we’re supposed to check you at 1:00’, but there isn’t anybody to check.

In regards to clinician factors, those in the High Acceptability group, made up of more physicians than the Low Acceptability group, more commonly spoke about lack of buy-in to the protocol from other clinicians.

Especially when we first started, there was a lot of grumbling … about treating patients the same, when they are different people.

Those in the Low Acceptability group were more likely to speak about patient resistance, rather than clinician resistance, to the protocol. These participants spoke about patients who, despite undergoing labor induction, wanted to let their body labor naturally or were distrustful of the interventions proposed.

There are some patients...that come with a birth plan, they come with a doula, and they are pushing back on treatment or exams.

Participants often described the culture of the included labor and delivery units as facilitative to protocol implementation.

I think our culture has some problems, but instituting protocols is not one of them. We are very used to that. I think our culture is also that, we want to improve outcomes for our patients, but sometimes we just feel a little helpless as to the best way to do that, because some of our patients do come in with a lot of co-morbidities. So, to have toolkits that we know actually do help our patients, I think we’re very willing to embrace.

#### Acceptability of current implementation strategies around the standardized labor induction protocol and suggestions for improved implementation

Many participants, particularly in the High Acceptability group, felt that they were well educated on the protocol going into implementation, and had the opportunity to ask questions with open discussion.

I think it made perfect sense… when I was presented with the information and the data, it was all sensible.

From both groups, participants agreed that nursing and resident trainee education around the protocol could be improved. In particular, there was focus on the number of residents rotating through labor and delivery, largely on a monthly basis, and that reminders were needed with rotation changes.

[In reference to nursing], I am sure there has been some education, but there should be even more. If there is education, about why we do [these induction steps], then I think some of the resistance to each step might be reduced.

I think constant rotation of residents makes it hard to implement a protocol, like they start every month, and then they forget that there’s even a protocol.

Participants from both groups agreed that better education of patients on the labor induction protocol is needed, with a focus on counseling about the protocol prior to admission for labor and delivery.

Well, I think education is really important … I think the nurses are doing a lot of education on the spot, but it would be great if the patients had more education about induction on the front end.

If there was something, some sort of handout, that would make it really easy.

When asked about the audit and feedback reports clinicians received regarding their fidelity to the protocol, participants all reported that they were both helpful and motivating to improve compliance.

I think that we’re driven by trying to be better and I think by seeing the things that we are not good at, it’s motivating to be better at them.

## Discussion

Measuring the acceptability of interventions accurately has proven to be difficult, despite its importance in implementation research. While the Acceptability of Intervention Measure is a validated and increasingly used measure in implementation science, the tendency for both interventions and their implementation strategies to receive high scores on the AIM assessment limits its utility. This study utilizes AIM in an innovative, explanatory sequential mixed-methods approach that harnesses participants with positive and negative responses to the AIM measure. This work was performed during the first year of a two year type I hybrid implementation-effectiveness study, and allowed our team to obtain a deep understanding of acceptability, both of the intervention itself (a standardized labor induction protocol), as well as the implementation strategies. As a result, we were able to develop concrete strategies targeted to improving acceptability to optimize the intervention and strategies for its implementation for year 2 of the parent trial.

In a study most similar to this work, Hoskins et al. evaluated parent acceptability of a S.A.F.E. Firearm Program in pediatric care. Here, a sequential mixed-methods approach was also used, beginning with the quantitative AIM survey, and finishing with semi-structured interviews with a convenience sample of respondents [17]. Their work also found uniformly high AIM scores. Our work expands upon the methodology set forth by Hoskins et al., interviewing deviant AIM samples rather than a convenience sample of survey respondents.

The notion of studying closest those with the most positive and/or negative attitudes towards a healthcare concept or intervention is not new. Positive deviance studies have focused on surveying and interviewing individuals successfully implementing interventions, with the goal of ascertaining valuable data to support others in similar implementation endeavors [10–12]. Similarly, negative deviance studies focus on those struggling to implement or unengaged with implementation, in order to catalog barriers and develop targeted responses [18]. Here, we focus on both positive and negative deviance in acceptability, to meet the objectives of both types of studies simultaneously.

In interviewing only those grouped into High (positive deviants) or Low Acceptability (negative deviants), we aimed to interview only those most data-rich respondents. As a result, we were able to develop 2 concrete strategies to improve acceptability in our work. The first focused on education. We initiated routine, multidisciplinary clinician re-education, to foster an understanding as well as community around protocol implementation. Next, we focused on involving patients as active participants prenatally. We developed a plan to educate patients, involving printed and electronic patient-centered information on labor induction, distributed to patients when scheduled for induction, as well as initiate ongoing conversations about labor induction and our methodologies throughout the third trimester, allowing patients to ask questions and prepare. We will evaluate the success of these implementation strategy adaptations in year 2 of our parent hybrid trial.

The strength of this study is in innovative measurement of acceptability using a mixed-methods approach, resulting in tangible changes to an ongoing implementation study. This study has several limitations. First, those who elected to complete the AIM survey may represent a self-selecting sample, which may already be biased towards High or Low Acceptability. Our methodology also assumes that the most data-rich individuals are those at both the extreme ends of acceptability, without evaluating the opinions of those in the Middle. We included clinicians from 2 sites, although both diverse and disparate from each other, which do not necessarily reflect the large variation in geography and practice model of labor and deliveries across the US.

## Conclusions

In conclusion, this sequential quantitative to qualitative methodology provided insightful data for the implementation outcome of acceptability around a standardized protocol for labor induction. The mixed-methods process described is likely applicable to assessing acceptability in other healthcare implementation endeavors, with benefits beyond either quantitative or qualitative evaluation alone. After application to other disciplines and interventions, this methodology may be refined and validated. Thus, we may be able to advance the scientific rigor and sophistication of the study of acceptability in implementation science, with the goal of eventually reaching a superior “gold standard” for acceptability evaluation.

## Data Availability

The datasets used and/or analyzed during the current study are available from the corresponding author on reasonable request.
